# Supplementary material for: Proteome Landscape during Ripening of Solid Endosperm from Two Different Coconut Cultivars Reveals Contrasting Carbohydrate and Fatty Acid Metabolic Pathway Modulation
Source: Int J Mol Sci. 2023 Jun 21;24(13):10431. doi: 10.3390/ijms241310431 (PMC10341993; doi:10.3390/ijms241310431)
Supplement: Supplementary file 1 [file ijms-24-10431-s001.zip › FIGURE LEGENDS FOR SUPPLEMENTARY MATERIAL.pdf]

FIGURE LEGENDS FOR SUPPLEMENTARY MATERIAL

Figure S1. Volume of water (mL) and fresh weight (g) during the ripening of two contrasting coconut varieties. Yucatan Green Dwarf (A) and Mexican Pacific Tall (B).

Figure S2. Visualization of principal component analysis based on the differences of normalized protein abundances among the different developmental stages and between YGD and MPT cultivars. Immature, intermediate and mature developmental stages from tall cultivar are located at the squares at right site of the figure, while those of the YGD cultivar are located at the squares in the left site of the figure.

Figure S3. Proteins of the glycolysis metabolic pathway determined in the YGD and MPT coconut varieties. A) glycolysis IV (plant cytosol); B) glycolysis II (from fructose 6-phosphate).

Table S1. Total proteins, core proteins, unique proteins and differentially accumulated proteins (DAPs) in the Yucatan Green Dwarf (YGD) and Mexican Pacific Tall (MPT) coconut varieties. For this analysis *Arabidopsis*, *Elaies guineensis* and *Phoenix dactylifera* protein homologs were used.

Table S2. Gene ontology enrichment and clustering of identified proteins in both Yucatan Green Dwarf (YGD), Mexican Pacific Tall (MPT), core or exclusive proteins in YGD or MPT. For this analysis, *Arabidopsis* protein homologs were used.
