# Supplementary material for: Proteome Landscape during Ripening of Solid Endosperm from Two Different Coconut Cultivars Reveals Contrasting Carbohydrate and Fatty Acid Metabolic Pathway Modulation
Source: Int J Mol Sci. 2023 Jun 21;24(13):10431. doi: 10.3390/ijms241310431 (PMC10341993; doi:10.3390/ijms241310431)
Supplement: Supplementary file 1 [file ijms-24-10431-s001.zip › Table S2.pdf]

Table S2. Gene ontology enrichment and clustering of identified proteins in both Yucatan green dwarf (YGD) and Mexican pacific tall (MPT) (Core) or exclusively in YGD or MPT. For this analysis we used *Arabidopsis* protein homologs.

Core

WARNING - This exported Revigo data is only useful for the specific purpose of constructing a TreeMap visualization.
Do not use this table as a general list of non-redundant GO categories, as it sets an extremely permissive
threshold to detect redundancies (c=0.10) and fill the 'representative' column, while normally c>=0.4 is recommended.
To export a reduced-redundancy set of GO terms, go to the Scatterplot or Table tab, and export from there.

TermID	Name	Frequency	Value	Uniqueness	Dispensability	Representative
GO:0000226	microtubule cytoskeleton organization	0.340003	-1.3678	0.988612	0.01040522	null
GO:0000278	mitotic cell cycle	0.386399	-1.3678	0.988494	0.01052856	null
GO:0005975	carbohydrate metabolic process	5.826191	-2.1907	0.933809	0.09069475	null
GO:0006096	glycolytic process	0.532551	-22.149	0.648503	0	null
GO:0006108	malate metabolic process	0.054243	-2.3475	0.884456	0.35081898	glycolytic process
GO:0010499	proteasomal ubiquitin-independent protein catabolic process	0.008394	-6.7258	0.843223	0.37012795	glycolytic process
GO:0030388	fructose 1,6-bisphosphate metabolic process	0.012265	-1.209	0.90986	0.36108737	glycolytic process
GO:0006457	protein folding	1.03808	-12.777	0.987502	0.01158996	null
GO:0006734	NADH metabolic process	0.056712	-1.4374	0.972475	0.0311262	null
GO:0007017	microtubule-based process	0.798415	-1.9585	0.987781	0.01208936	null

GO:0015 977	carbon fixation	0.0503 02	- 1.2 859	0.962 67	0.0445 3891	null
GO:0015 979	photosynthesis	0.2175 87	- 1.4 538	0.948 841	0.0609 9619	null
GO:0045 454	cell redox homeostasis	0.1778 77	- 2.7 017	0.982 087	0.0098 2244	null
GO:0032 889	regulation of vacuole fusion, non-autophagic	0.0006 23	- 1.8 008	0.992 874	0.1356 3283	cell redox homeostasis
GO:0046 686	response to cadmium ion	0.0088 23	- 41. 215	0.804 765	0	null
GO:0009 408	response to heat	0.1714 54	- 5.7 167	0.767 998	0.2115 2305	response to cadmium ion
GO:0009 615	response to virus	0.1706 13	- 1.7 198	0.859 91	0.2606 9386	response to cadmium ion
GO:0046 685	response to arsenic-containing substance	0.0184 7	- 1.0 885	0.815 745	0.3573 0895	response to cadmium ion
GO:0051 085	chaperone cofactor-dependent protein refolding	0.0167 25	- 8.2 175	0.961 239	0.0081 5552	null

Only Yucatan green dwarf (YGD)

WARNING - This exported Revigo data is only useful for the specific purpose of constructing a TreeMap visualization.
Do not use this table as a general list of non-redundant GO categories, as it sets an extremely permissive
threshold to detect redundancies (c=0.10) and fill the 'representative' column, while normally c>=0.4 is recommended.
To export a reduced-redundancy set of GO terms, go to the Scatterplot or Table tab, and export from there.

TermID	Name	Frequ ency	Valu e	Uniqu eness	Dispen sability	Representative
GO:000 1558	regulation of cell growth	0.072 13	- 1.28 418	1	0	null
GO:000 6457	protein folding	1.038 08	- 7.60 033	0.988 23374 5	0	null
GO:000 6633	fatty acid biosynthetic process	0.750 35	- 3.28 483	0.747 44286	0.0120 1001	null

GO:0009298	GDP-mannose biosynthetic process	0.03738	-1.75374	0.851718388	0.24996828	fatty acid biosynthetic process
GO:0006890	retrograde vesicle-mediated transport, Golgi to endoplasmic reticulum	0.06958	-1.00604	1	0	null
GO:0007017	microtubule-based process	0.79842	-1.06986	0.988473525	0.01208936	null
GO:0009793	embryo development ending in seed dormancy	0.0213	-2.00587	1	0	null
GO:0042026	protein refolding	0.09236	-3.93554	0.931447121	0.00983271	null
GO:0046686	response to cadmium ion	0.00882	-17.5331	0.788621006	0	null
GO:0009408	response to heat	0.17145	-7.92082	0.777939937	0.21152305	response to cadmium ion
GO:0009615	response to virus	0.17061	-2.20337	0.855659051	0.26069386	response to cadmium ion

Only Mexican pacific tall (MPT)

WARNING - This exported Revigo data is only useful for the specific purpose of constructing a TreeMap visualization.
Do not use this table as a general list of non-redundant GO categories, as it sets an extremely permissive
threshold to detect redundancies (c=0.10) and fill the 'representative' column, while normally c>=0.4 is recommended.
To export a reduced-redundancy set of GO terms, go to the Scatterplot or Table tab, and export from there.

TermID	Name	Frequency	Value	Uniqueness	Dispensability	Representative
GO:0005975	carbohydrate metabolic process	5.82619	-1.82799	0.928665635	0.09037153	null
GO:1901135	carbohydrate derivative metabolic process	6.81372	-1.06903	0.938344521	0.10335243	carbohydrate metabolic process

GO:0006099	tricarboxylic acid cycle	0.51738	-4.47625	0.863839013	0	null
GO:0006457	protein folding	1.03808	-2.4677	0.98771216 1	0.01232809	null
GO:0007010	cytoskeleton organization	0.95778	-3.07109	0.98779347	0.01146205	null
GO:0009793	embryo development ending in seed dormancy	0.0213	-1.81056	0.946053188	0	null
GO:0015031	protein transport	2.72571	-1.07077	1	0	null
GO:0015979	photosynthesis	0.21759	-1.28127	0.943424156	0.06084153	null
GO:0033356	UDP-L-arabinose metabolic process	0.00333	-1.43414	0.833894742	0.04703988	null
GO:0006097	glyoxylate cycle	0.05384	-1.36853	0.816413507	0.26865503	UDP-L-arabinose metabolic process
GO:0006101	citrate metabolic process	0.01085	-1.36853	0.822741841	0.16439007	UDP-L-arabinose metabolic process
GO:0006536	glutamate metabolic process	0.20235	-1.21764	0.784556398	0.3259023	UDP-L-arabinose metabolic process
GO:0015995	chlorophyll biosynthetic process	0.04778	-1.23061	0.874933866	0.1328704	UDP-L-arabinose metabolic process
GO:0035999	tetrahydrofolate interconversion	0.15891	-1.0482	0.83996568 1	0.20520025	UDP-L-arabinose metabolic process
GO:0042744	hydrogen peroxide catabolic process	0.12272	-1.64202	0.848247804	0.05792981	null
GO:0046686	response to cadmium ion	0.00882	-23.	0.806812261	0	null

			0645			
GO:0009651	response to salt stress	0.03027	-2.06823	0.812330435	0.19048836	response to cadmium ion
GO:0080148	negative regulation of response to water deprivation	0.00018	-1.51198	0.986854261	0	null
GO:0045454	cell redox homeostasis	0.17788	-1.45972	0.969251392	0.11669074	negative regulation of response to water deprivation
GO:0051252	regulation of RNA metabolic process	10.1765	-1.73115	0.978347648	0.28832307	negative regulation of response to water deprivation
