# Supplementary figures and images for: Proteome Landscape during Ripening of Solid Endosperm from Two Different Coconut Cultivars Reveals Contrasting Carbohydrate and Fatty Acid Metabolic Pathway Modulation
Source: Int J Mol Sci. 2023 Jun 21;24(13):10431. doi: 10.3390/ijms241310431 (PMC10341993; doi:10.3390/ijms241310431)

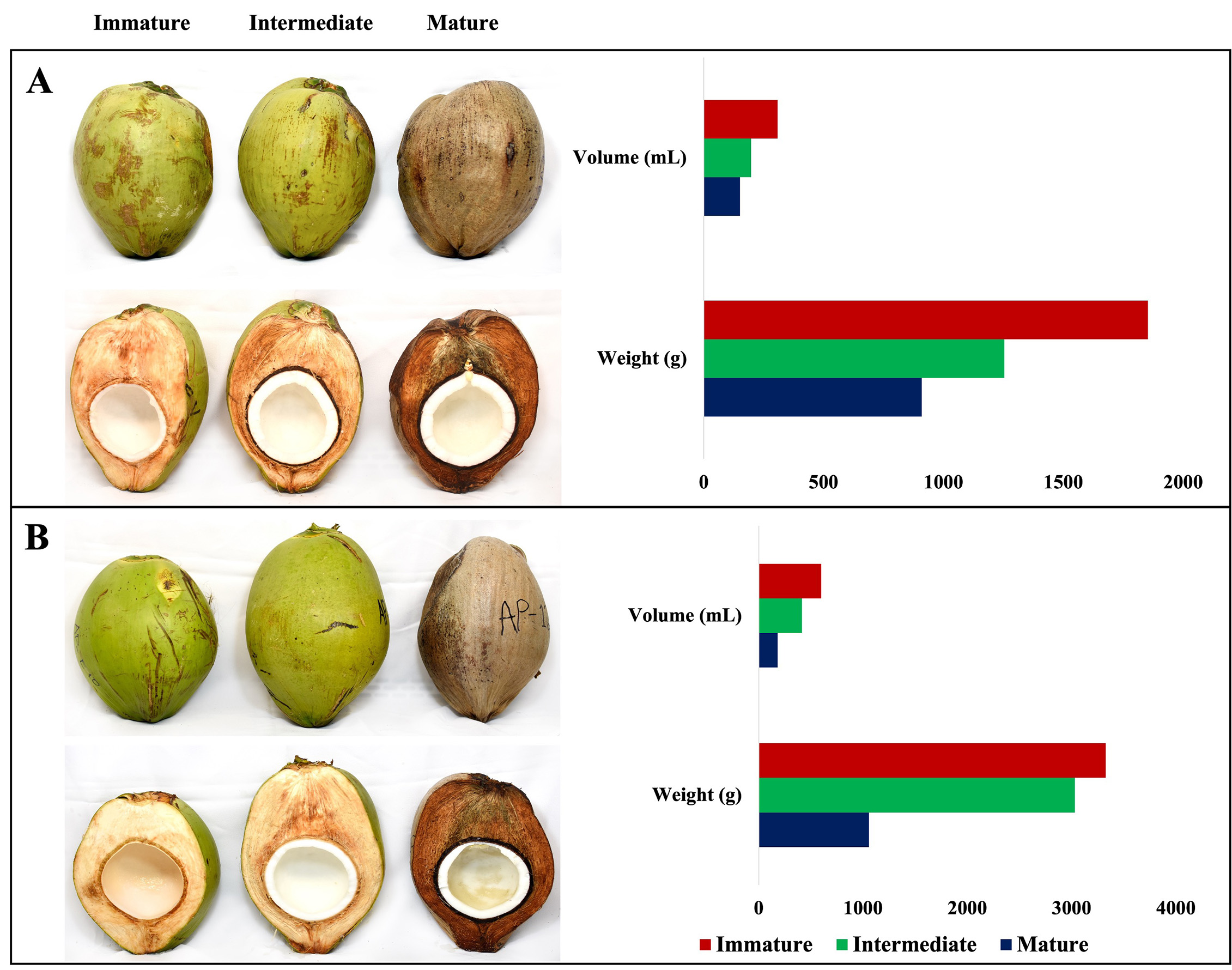

Supplement: Supplementary file 1 [file ijms-24-10431-s001.zip › Figure S1.tif]

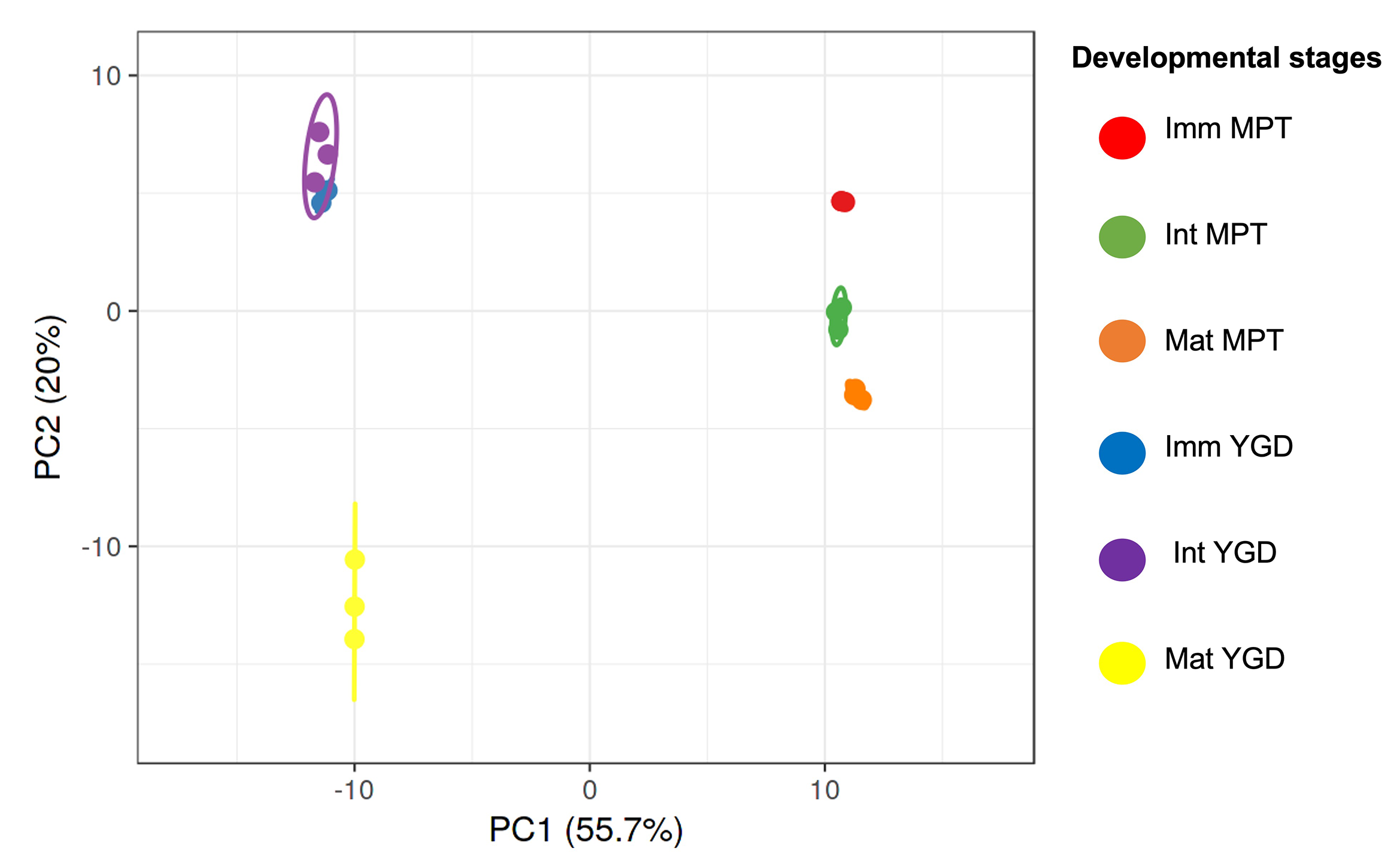

Supplement: Supplementary file 1 [file ijms-24-10431-s001.zip › Figure S2.tif]

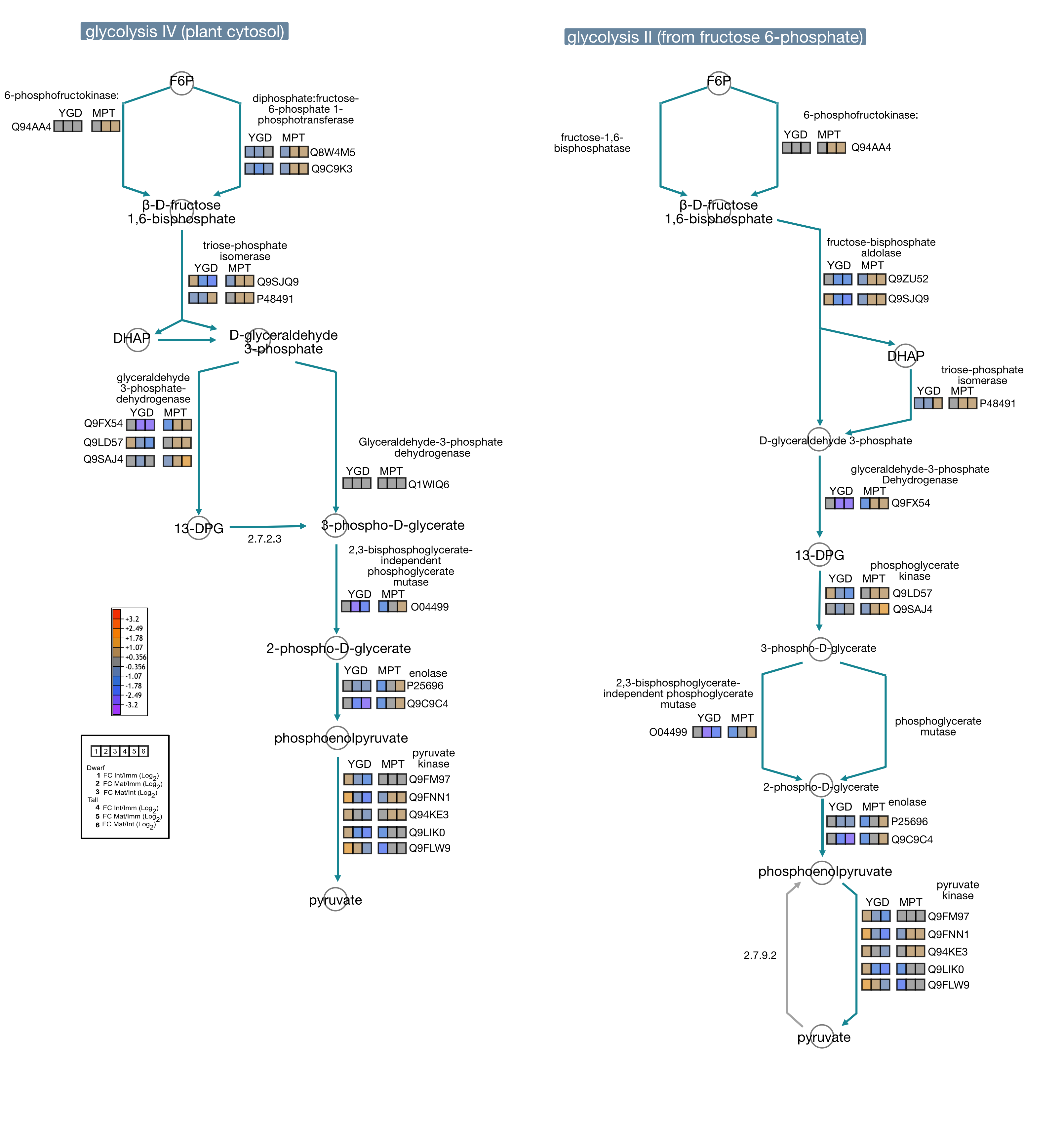

Supplement: Supplementary file 1 [file ijms-24-10431-s001.zip › Figure S3.tiff]
